# Association of GH/*HaeIII* polymorphism with growth and developmental traits of Morkaraman sheep

**DOI:** 10.5194/aab-68-575-2025

**Published:** 2025-09-24

**Authors:** Ulku Dagdelen, Nurinisa Esenbuga

**Affiliations:** 1 Department of Animal Science, Faculty of Agriculture, Atatürk University, Erzurum 25240, Türkiye

## Abstract

This study investigated the impact of GH gene polymorphism in Morkaraman ewes on placental traits and lamb development characteristics across three years (2020–2022) at Atatürk University Sheep Farm, Erzurum. PCR–RFLP method with *HaeIII* endonuclease was used to identify GH gene polymorphism. Two genotypes were found in Morkaraman sheep: AA (50.40 %) and AB (49.60 %). Ewes with AA and AB genotypes showed birth weights of 4.32 and 4.43 kg, weaning weights of 14.32 and 15.42 kg, and daily gains of 209 and 223 g, respectively. While GH genotype did not significantly affect birth weight, it significantly influenced weaning weight ( p<0.01 ) and daily live weight gain ( p<0.05 ). Placental characteristics examined included placental weight, surface area, efficiency, total cotyledon numbers, cotyledon length and width, cotyledon efficiency, total cotyledon weight, and density. Genotype showed no significant effect on placental traits, but parity significantly affected placental area ( p<0.05 ). While lamb sex had no impact, delivery type significantly influenced placental weight ( p<0.05 ). Genotype showed highly significant effects ( p<0.01 ) on total cotyledon area, weight, and density among cotyledon characteristics. The present study found that Morkaraman ewes with the AB genotype of the GH gene revealed superior growth and placental characteristics, with their lambs also exhibiting enhanced growth traits. The results of the study indicate that beneficial genotypes can be identified from birth, thus leading to the development of improved breeding strategies.

## 1 Introduction

Growth and development in mammals represent intricate biological processes that encompass both prenatal and postnatal phases, orchestrated through complex interactions between genetic, hormonal, and environmental factors. The prenatal period is characterized by embryonic development and fetal growth under tight genetic control, while postnatal growth is additionally modulated by environmental conditions and management practices (Akçapınar, 2000). This developmental trajectory is regulated by a sophisticated endocrine network, with the growth hormone (GH) serving as a central mediator of skeletal development, cellular proliferation, and muscle function (Ouchar, 2019; Lacroix et al., 2002).

The synthesis and secretion of GH, regulated by hypothalamic growth hormone-releasing hormone (GHRH), exert pleiotropic effects on protein biosynthesis across diverse tissue types (Gorlov et al., 2014; Malewa et al., 2014; Othman et al., 2015). Genetic variations in the GH gene, identifiable through PCR–RFLP analysis using the *HaeIII* enzyme, may influence these physiological processes and subsequent phenotypic expressions.

During pregnancy, the placenta emerges as a crucial temporary organ that not only facilitates nutrient exchange and waste elimination but also functions as an endocrine organ (Igwebuike, 2010). Notably, placental GH production peaks between days 35 and 50 of gestation, coinciding with maximal placental development, although it constitutes only 1 % of total GH production compared to pituitary secretion (Lacroix et al., 2002).

The Morkaraman sheep, indigenous to Eastern Anatolia, represents a significant genetic resource adapted to the region's continental climate (Türkyılmaz and Esenbuğa, 2022). Characterized by medium to large frame size and distinctive fat-tail morphology (Yilmaz et al., 2013), this breed exhibits notable maternal traits with an average prolificacy of 1.05–1.15 lambs per ewe (Türkyılmaz, 2014). Their genetic diversity and adaptation to local conditions make them particularly valuable for both economic and cultural aspects of traditional farming in Eastern Anatolia (Özay, 2019).

This study aims to elucidate the relationship between GH/*HaeIII* gene polymorphism and growth characteristics in Morkaraman ewes through three primary objectives: characterization of GH/*HaeIII* gene polymorphism patterns; assessment of polymorphism effects on key growth parameters including birth weight, weaning weight, and daily body weight gain; and evaluation of the broader implications of these genetic variations on development traits.

Understanding the association between GH gene polymorphisms and growth parameters could significantly enhance selective breeding strategies in Morkaraman sheep. Moreover, this research has potential implications for both practical animal husbandry and future biotechnological applications while contributing to the fundamental understanding of ovine genetics and reproductive biology.

## 2 Material and methods

### 2.1 Material

#### 2.1.1 Resource population

This investigation encompassed a comprehensive dataset comprising 123 purebred Morkaraman ewes and their progeny ( n=388 lambs) from three consecutive breeding seasons (2020–2022) at the Sheep Breeding Unit of the Atatürk University Food and Livestock Application and Research Centre. The maternal cohort was stratified according to lactation parity (second, third, and fourth or greater parities). The longitudinal breeding program yielded 124 lambs in 2020, 129 in 2021, and 135 in 2022, demonstrating consistent reproductive performance across the study period.

#### 2.1.2 Feeding management

Lambs were housed with their mothers after birth. When lambs reached the 60th day, they became weaned. After weaning, lambs grazed on pasture were given supplementary feeding at the rate of 2 % of their live weight. The nutritional regimen consisted of two primary components: roughage and concentrate feed. The roughage component comprised dry meadow grass (96 % dry matter) sourced from Atatürk University's Plant Production Application and Research Centre, with the following nutritional composition: crude protein (6 %), crude fat (2.5 %), crude fiber (33 %), and metabolizable energy (1800 kcal kg^−1^). This roughage was provided ad libitum throughout the study period.

The concentrate feed, obtained from a commercial feed manufacturer, was formulated to contain 14 % crude protein, 2 % crude fat, 13 % crude fiber, and 9 % crude ash, with mineral supplementation including Na (0.35 %) and Mg (0.20 %). The concentrate was fortified with vitamins A (15 000 IU), D3 (3000 IU), and E (30 mg IU^−1^), providing 2750 kcal kg^−1^ of metabolizable energy. A strategic flushing protocol was implemented for the entire flock preceding the breeding season. The detailed formulation and chemical composition of the experimental diets are presented in Table 1.

**Table 1:** Formulation and chemical composition of experimental diet.

Ingredients	CON
Barley	464.3
Wheat bran	78.6
Soybean meal	157.1
Dicalcium phosphate	7.1
Sodium chloride	7.1
Meadow grass	285.8
Chemical composition	CON
Dry matter	87.7
Crude protein	12.4
Ether extract	2.3
Crude fiber	17.8
Ash	9.0
Starch	27.0
Ca	0.26
Metabolizable energy	CON
MJ kg^−1^	10.6

### 2.2 Methods

#### 2.2.1 Registration of births and collection of placenta samples

A systematic protocol was implemented for parturition monitoring and data collection. Parturient ewes were transferred to individual maternity pens to minimize disturbance during the birthing process. Immediately postpartum, comprehensive data collection was performed, including lamb birth weight measurements using a calibrated precision scale ( ±50 g accuracy). Each neonate was assigned a unique identification number, and relevant peripartum data were documented, encompassing parturition date, maternal age, offspring sex, and birth type.

**Table 2:** Parameters used for the determination of placental characteristics and their definitions.

Parameters	Calculation methods
Placenta weights (g) (PWs)	Weighing the placenta after cleaning it from all fluids and materials such as straw, feces, and grass.
Placenta area (cm^2^) (PA)	Placenta width × placenta length (Image J-Lab Program)
Placenta efficiency (PE)	Lamb birth weight/placenta weight
Total number of cotyledons (TCNs)	Total number of cotyledons on the placenta
Cotyledon length and width (CL and CW)	Transverse and longitudinal lengths of cotyledons (Image J-Lab Program)
Cotyledon efficiency (CE)	Lamb birth weight/total cotyledon area
Total cotyledon weights (TCWs)	Weighting of cotyledons after they leave the placenta
Cotyledon density (CD)	Number of cotyledons/placenta weight
Total cotyledon area (cm^2^) (TCA)	π× width of cotyledon × length of cotyledon /2 (Image J-Lab Program)

Following placental expulsion, the specimens were collected in 2022, cleaned of debris, and labeled with corresponding maternal identification. To maintain sample integrity and minimize degradation, placental analysis was conducted on the same day of collection in the Animal Husbandry Laboratory. Each placenta underwent gravimetric analysis using standardized protocols given in Table 2. Placenta weights, placenta area, placenta productivity, total number of cotyledons, cotyledon length and width, cotyledon productivity, total cotyledon weights, cotyledon density and total cotyledon area were determined.

#### 2.2.2 Blood collection from animals and DNA isolation

All experimental procedures were conducted in accordance with the institutional animal care guidelines and approved by the Atatürk University Animal Experiments Local Ethics Committee (ATA-HADYEK, approval no. 11/157, dated 30 November 2020).

Blood samples were collected from Morkaraman ewes ( n=123 ) at 2 months postpartum via jugular venipuncture using sterile vacutainers containing K_3_EDTA (ethylenediaminetetraacetic acid) as an anticoagulant. Each sample was uniquely identified with the corresponding ovine identification number. Samples were transported under controlled temperature conditions to the Molecular Genetics Laboratory, Department of Animal Husbandry, Faculty of Agriculture, Atatürk University.

Genomic DNA extraction was performed using the Qiagen Genomic DNA Purification Kit (Qiagen Inc., Chatsworth, CA, USA) following the manufacturer's standardized protocol. DNA quality and concentration were assessed spectrophotometrically using a NanoDrop 1000 spectrophotometer (Thermo Scientific, Waltham, MA, USA). The extracted DNA was standardized to a working concentration of 50 ng $µL^{-1}$ and stored at 4 °C. For samples not meeting the predetermined quality parameters, DNA isolation procedures were repeated to ensure optimal template quality for subsequent analyses.

**Table 3:** Primer of the GH region amplified by PCR.

Gene	Primer sequence	Annealing temperature	Product size	Reference
GH F	CTCTGCCTGCCCTGGACT	62°	422 bp	Hua et al. (2009)
GH R	GGAGAAGCAGAAGGCAACC			

#### 2.2.3 PCR and RFLP analyses

The primer pairs for the sheep GH gene were as follows: forward primer is CTCTGCCTGCCCTGGACT, and the reverse primer is GGAGAAGCAGAAGGCAACC, given in Table 3 (Hua et al., 2009). A reaction mixture of 25.2 µL was used for performing PCR reaction, and it consisted of ddH2O 16.8 µL , 2.5 µL of 10× buffer, 1 µL (10 pmol) of each primer, 0.5 µL of dNTPS, 1.2 µL of MgCI_2_, 0.2 µL of Taq DNA polymerase (Bio-Labs, M0273L), and 2 µL (50–100 $\mathrm{ng}\;µL^{-1}$ ) of DNA template. The contents were thoroughly mixed by vortexing followed by PCR amplification in a thermal cycler (BIO-RAD T100) after standardizing the PCR parameters.

The PCR protocol consisted of an initial denaturation step at 94 °C for 2 min, followed by 34 cycles of denaturation at 94 °C for 45 s, annealing at 59 °C for 45 s, and an extraction step at 72 °C for 45 s, followed by a final extension step at 72 °C for 7 min. Resulting PCR product was checked on 2 % agarose gel, including 0.5 $µg\;{\mathrm{mL}}^{-1}$ of ethidium bromide for validation of the amplified product.

RFLP analysis was conducted for identifying the polymorphism at a targeted locus. Restriction endonuclease *HaeIII* (Thermo Scientific Inc.) was used for digestion of resulting PCR products. The incubation of the PCR products was carried out for a period of 10–12 h at a temperature of 37 °C in a final volume of 20 µL , including 8–10 µL of the PCR product, 5 µL of the buffer R, 2.5 µL of the buffer Tango, and 6 µL *HaeIII* restriction enzyme. The digested PCR products were run to 2 %–2.5 % agarose gel electrophoresis stained with ethidium bromide (500 $µ\;{\mathrm{mL}}^{-1}$ in H_2_O) up to 2.5 h, and the digested PCR products were genotyped under UV light. Determination of genotype results in imaging after enzyme digestion is given in Table 3.

#### 2.2.4 Statistics

After imaging for genotyping DNA bands belonging to the GH gene region obtained after electrophoresis cutting, firstly analyses were performed in terms of population parameters. As a result of the analyses performed, the genotype and allele frequencies of the groups were determined according to both population and environmental conditions. In line with the obtained data, whether the studied population was in Hardy–Weinberg equilibrium was determined by the chi-square ( χ 2) test.

In the variance analysis of the data regarding GH gene polymorphisms, growth and placental characteristics obtained in the study, SPSS 20.0 (2010) package program GLM procedure was used; differences between groups were determined by a Duncan multiple comparison test.

The mathematical model is as follows: $Y_{ijklm}=\mu +a_{i}+b_{j}+c_{k}+d_{l}+e_{ijklm}$ , where $Y_{ijklm}$ is the growth and placental characteristics; μ is the expected mean; $a_{i}$ is the effect of the i th genotype ( i=1 , 2; AA, AB); $b_{j}$ is the effect of j th parity ( j=1 , 2, 3; second, third, fourth and > parity); $c_{k}$ is the effect of k th lamb sex ( k=1 , 2; male, female); $d_{l}$ is the effect of l th birth type ( l=1 , 2; single, twin); and $e_{ijklm}$ is the random error.

## 3 Results

### 3.1 GH genotypes

In the present study, AA and AB genotypes were identified. BB genotype was not found in the study. In terms of allele frequencies, the frequency of allele A was higher than the frequency of allele B. The GH genotype and allele frequencies obtained in the study are presented in Table 5 in order of lactation.

**Table 4:** Genotype results to be determined in imaging after enzyme cutting.

GH genotypes	Bands (base pairs)
AA	366 and 56
AB	422, 366, and 56
BB	422

**Table 5:** Parity and population distribution of genotype and allele frequencies in terms of GH gene region.

Lactation order		Genotype frequencies		Allele frequencies	
		AA	AB	A	B
General	N	62	61	185	61
	%	50.40	49.60	0.752	0.248
2	N	21	16	58	16
	%	56.8	43.2	0.784	0.216
3	N	26	12	64	12
	%	68.4	31.6	0.842	0.158
≥4	N	15	33	63	33
	%	31.3	68.8	0.656	0.344

**Table 6:** Observed and expected values of genotypes in terms of GH gene region and chi-square ( χ 2) test analysis results.

	Observed		Expected		Chi square
	AA	AB	AA	AB	( χ 2)
General	62	61	62.1	60.9	
Parity					
2	21	16	18.7	18.3	**
3	26	12	19.2	18.8	
≥4	15	33	24.2	23.8	

** Very significant ( p<0.01 ).

**Table 7:** Growth characteristics of Morkaraman sheep according to their GH genotypes.

	N	AA	AB	P
Birth weight	123	4.32±0.08	4.43±0.08	ns
Wean weight	123	14.32±0.25	15.42±0.25	**
Daily weight gain	123	0.209±0.004	0.223±0.005	*

* Significant ( p<0.05 ). ** Very significant ( p<0.01 ); ns: insignificant.

The observed and expected values of the genotypes in terms of the GH gene region and the chi-square ( χ 2) analysis results are given in Table 6. According to the Hardy–Weinberg equilibrium test results of the study, it was determined that it was not in equilibrium ( p<0.01 ).

### 3.2 Growth characteristics of sheep

Growth characteristics of Morkaraman sheep according to GH genotypes are given in Table 7. According to the results obtained, although it was determined that GH genotype had no statistical effect on birth weight, it was observed that the birth weight of lambs born from ewes with AB genotype was relatively higher than lambs born from ewes with AA genotype.

The study showed that GH gene polymorphisms had a very significant effect on weaning weight ( p<0.01 ) and a significant effect on daily live weight gain ( p<0.05 ). In terms of results, birth weight was found to be statistically similar in AB genotype ewes but relatively higher in AA genotype ewes.

**Table 8:** Average birth weight of lambs born in different years from Morkaraman sheep in terms of genotype, parity, lamb sex, and birth type.

	Year 1 (2019)	Year 2 (2020)	Year 3 (2021)	P
	n=124	n=129	n=135	
Genotype	*	ns	ns	
AA	4.13 ${}^{b}\pm 0.14$	5.08 ${}^{a}\pm 0.10$	4.57 ${}^{b}\pm 0.11$	**
AB	4.56 ${}^{b}\pm 0.11$	4.98 ${}^{a}\pm 0.12$	4.66 ${}^{b}\pm 0.15$	*
Parity	ns	ns	ns	
1	4.00±0.15			
2	4.52±0.19	5.06±0.16		ns
3	4.57 ${}^{b}\pm 0.22$	5.15 ${}^{a}\pm 0.16$	4.58 ${}^{b}\pm 0.10$	**
4	4.36 ${}^{b}\pm 0.23$	5.08 ${}^{a}\pm 0.21$	4.63 ${}^{b}\pm 0.10$	**
5+		4.99±0.20	4.62±0.14	ns
Lamb sex	ns	*	ns	
Male	4.34 ${}^{b}\pm 0.15$	5.25 ${}^{a}\pm 0.12$	4.63 ${}^{b}\pm 0.16$	**
Female	4.30 ${}^{b}\pm 0.12$	4.76 ${}^{a}\pm 0.09$	4.59 ${}^{b}\pm 0.09$	*
Birth type	**	*	*	
Single	4.44 ${}^{b}\pm 0.10$	5.08 ${}^{a}\pm 0.08$	4.67 ${}^{b}\pm 0.10$	**
Twin	3.37 ${}^{b}\pm 0.34$	4.17 ${}^{a}\pm 0.18$	4.26 ${}^{a}\pm 0.13$	**

* Significant ( p<0.05 ). ** Very significant ( p<0.01 ); ns: insignificant. ^a,b^ The difference between means indicated by the same letter between columns is insignificant.

Average birth weights of lambs born in different years from Morkaraman sheep in terms of genotype, parity, lamb sex, and birth type are shown in Table 8. In terms of birth weight, lambs born from ewes with AB genotype had significantly ( p<0.05 ) higher values than AA genotype in the first year, while there was no significant difference between the genotypes in the second and third years. The birth weight of lambs obtained from AA- and AB-genotyped ewes varied in different years. The highest lamb birth weight was obtained from the lambs born in the second year of the ewes in the third parity, while the lowest lamb birth weight was obtained from the lambs born from the animals in the first parity. Lambs born in third and fourth parity ewes in different years had significantly ( p<0.01 ) higher birth weights. According to the results obtained, it was determined that year had a highly significant effect ( p<0.01 ) on birth weight of male lambs and a significant effect ( p<0.05 ) on birth weight of female lambs. In the study, it was determined that sex had a highly significant effect ( p<0.01 ) on the birth weight of male lambs obtained in the second year, but no effect was found on the birth weight of female lambs born in the first and third years. It was determined that the birth weight of male lambs was higher than the birth weight of female lambs in all years regardless of the year of birth. In terms of birth type, the birth weight of single lambs was found to be significantly ( p<0.01 ) higher than twin lambs. Regardless of the year of birth, the birth weight of single lambs was higher than the birth weight of twin lambs.

**Table 9:** Average weaning weight of lambs born in different years of Morkaraman sheep in terms of genotype, parity, lamb sex, and birth type.

	Year 1 (2019)	Year 2 (2020)	Year 3 (2021)	P
	n=124	n=129	n=135	
Genotype	*	**	*	
AA	15.80 ${}^{b}\pm 0.90$	19.12 ${}^{a}\pm 0.58$	19.31 ${}^{a}\pm 0.63$	*
AB	17.71 ${}^{b}\pm 0.61$	21.25 ${}^{a}\pm 0.47$	20.88 ${}^{a}\pm 0.56$	**
Parity	ns	*	*	
1	15.72±0.56			
2	17.53±1.29	19.66 ${}^{B}\pm 0.60$		ns
3	16.89 ${}^{b}\pm 0.97$	21.45 ${}^{\mathrm{Aa}}\pm 0.63$	20.81 ${}^{\mathrm{Aa}}\pm 0.62$	**
4	17.56 ${}^{b}\pm 1.57$	21.84 ${}^{\mathrm{Aa}}\pm 0.81$	21.69 ${}^{Aa}\pm 0.68$	*
5+		19.38 ${}^{B}\pm 0.88$	19.81 ${}^{B}\pm 0.97$	ns
Lamb sex	**	**	*	
Male	18.66 ${}^{b}\pm 0.76$	21.60 ${}^{a}\pm 0.44$	21.04 ${}^{a}\pm 0.59$	**
Female	15.76 ${}^{b}\pm 0.72$	19.50 ${}^{a}\pm 0.52$	20.11 ${}^{a}\pm 0.52$	**
Birth type	*	**	ns	
Single	16.02 ${}^{b}\pm 0.50$	21.14 ${}^{a}\pm 0.35$	20.72 ${}^{a}\pm 0.43$	**
Twin	18.33±1.27	17.29±0.73	20.13±1.16	ns

* Significant ( p<0.05 ). ** Very significant ( p<0.01 ); ns: insignificant. ^a,b^ The difference between means denoted by the same letter between year is insignificant. ^A,B^ The difference between means indicated by the same letter between parity is insignificant.

Average weaning weights of lambs born in different years of Morkaraman sheep in terms of genotype, parity, lamb sex and birth type are presented in Table 9. According to the values obtained as a result of the study, the effect of year on weaning weight of lambs obtained from ewes with AB genotype was very significant ( p<0.01 ), while the effect of year on lambs obtained from ewes with AA genotype was significant ( p<0.05 ). According to the results, it was determined that lambs obtained from ewes with AB genotype had significantly ( p<0.05 ) higher weaning weights in the first and third years and very significantly ( p<0.01 ) higher weaning weights in the second year. It was determined that the highest weaning weight was reached in lambs born in the second year from ewes with AB genotype. In parallel with the fact that AB-genotyped individuals were relatively higher in terms of birth weight than AA-genotyped individuals, weaning weight was found to be statistically higher. The effect of birth type on weaning weight was found to be significant ( p<0.05 ) in year 1, very significant ( p<0.01 ) in year 2, and insignificant in year 3. As a result of the study, weaning weights of single lambs born in the second and third years were found to be significantly ( p<0.01 ) higher, while the effect of year was found to be insignificant in twin lambs. In terms of year, birth and weaning weights of lambs born in the second year were found to be higher than in other years. In parallel with the fact that AB-genotyped individuals were relatively higher in terms of birth weight than AA-genotyped individuals, weaning weight was found to be statistically higher. In this study, it was observed that genotype had an effect on weaning weight, and it is thought that determining the GH gene polymorphism in order to determine the weaning weights of the lambs to be marketed at an early age and keeping the lambs with AB genotype will provide a predictable profitability for the enterprise.

**Table 10:** Placenta characteristics of Morkaraman sheep.

	Placenta area	Placenta weights	Placenta efficiency
	(cm^2^)	(g) (PWs)	(BWs/PWs)
Genotype of dam	ns	ns	ns
AA	1998.47±207.21	465.30±40.38	10.75±1.38
AB	2143.06±198.28	493.72±38.64	10.76±1.32
Parity	*	ns	ns
2	2330.84 ${}^{A}\pm 197.88$	459.46±38.56	11.61±1.31
3	2329.01 ${}^{A}\pm 206.74$	510.57±40.29	9.60±1.37
4≥	1552.44 ${}^{B}\pm 299.43$	468.50±58.35	11.05±1.99
Lamb sex	ns	ns	ns
Female	2260.41±186.22	469.08±36.29	10.16±1.49
Male	1881.12±224.96	489.94±43.84	11.35±1.24
Birth type	ns	*	ns
Twin	2012.70±298.09	556.09±58.09	9.63±1.98
Single	2128.82±122.49	402.94±23.87	11.88±0.81
GenxP	ns	ns	ns
GenxLS	*	ns	ns
GenxBT	ns	ns	ns
PxLS	ns	ns	ns
PxBT	ns	ns	ns
BTxLS	ns	**	**

* Significant ( p<0.05 ). ** Very significant ( p<0.01 ); ns: insignificant. ^A,B^ The difference between means indicated by the same letter between columns is insignificant. Gen: genotype; P: parity; LS: lamb sex; BT: birth type; BW: birth weight; PW: weight of placenta.

### 3.3 Placenta characteristics of Morkaraman sheep

Placenta characteristics of Morkaraman sheep are given in Table 10. The effect of parity on placenta area was found to be statistically significant ( p<0.01 ). According to the results obtained in the study, placenta areas of ewes in the second and third lactation were found to be higher than those of ewes in the fourth lactation. In the study, it was determined that the type of birth had a statistically very significant ( p<0.01 ) effect on placenta weight. It was determined that genotype had a highly significant ( p<0.01 ) effect on total cotyledon area, cotyledon weight, and cotyledon density. Statistically examined, it was determined that AB genotype sheep had higher values than AA genotype sheep in terms of total cotyledon area, cotyledon weight, and cotyledon density. In the study, it was determined that parity had a significant effect on cotyledon number, cotyledon efficiency, and cotyledon density ( p<0.05 ) and a highly significant effect on cotyledon weight ( p<0.01 ).

**Table 11:** General cotyledon characteristics in Morkaraman sheep.

	Total cotyledon	Cotyledon	Cotyledon	Number of	Cotyledon	Cotyledon	Cotyledon
	area	length	width	cotyledons	weight	activity	density
	(cm^2^)	(cm)	(cm)	(TCN)	(g)	(BWs/TCAs)	(CN/PWs)
Genotype	**	ns	ns	ns	**	ns	**
AA	364.96±27.99	3.04±0.12	2.52±0.10	60.59±4.21	50.87±7.75	35.02±5.69	0.155±0.021
AB	371.96±27.30	2.85±0.11	2.36±0.10	69.08±4.11	53.47±7.56	22.26±5.55	0.165±0.021
Parity	ns	ns	ns	*	**	*	*
2	345.17±27.78	2.82±0.11	2.34±0.10	65.12 ${}^{A}\pm 4.18$	50.63 ${}^{B}\pm 7.70$	31.29 ${}^{A}\pm 5.64$	0.170 ${}^{A}\pm 0.021$
3	395.92±28.14	3.05±0.12	2.50±0.10	67.11 ${}^{A}\pm 4.24$	53.55 ${}^{A}\pm 7.80$	26.10 ${}^{B}\pm 5.72$	0.150 ${}^{B}\pm 0.021$
4≥	364.29±38.83	2.96±0.16	2.46±0.14	62.27 ${}^{B}\pm 5.84$	52.34 ${}^{A}\pm 10.76$	28.54 ${}^{A}\pm 7.89$	0.157 ${}^{B}\pm 0.029$
Lamb sex	**	*	*	*	*	*	**
Female	365.33±25.66	2.89±0.11	2.37±0.09	66.80±3.86	48.03±7.11	31.71±5.21	0.164±0.019
Male	371.60±30.41	2.99±0.13	2.50±0.11	62.87±4.58	56.31±8.42	25.57±6.18	0.156±0.023
Birth type	ns	ns	ns	**	*	ns	**
Twin	439.87±42.57	3.21±0.18	2.63±0.15	64.79±6.41	57.33±11.79	19.04±8.65	0.155±0.032
Single	297.06±15.71	2.68±0.06	2.24±0.05	64.87±2.36	47.01±4.35	38.25±3.19	0.165±0.012
GenxP	ns	**	*	ns	*	**	ns
GenxLS	**	*	**	**	**	**	**
GenxBT	ns	ns	ns	**	*	**	**
PxLS	*	*	*	*	**	**	*
PxBT	**	ns	ns	ns	*	*	ns
BTxLS	ns	ns	ns	*	ns	*	ns

* Significant ( p<0.05 ). ** Very significant ( p<0.01 ); ns: insignificant. ^A,B^ The difference between means indicated by the same letter between columns is insignificant. Gen: genotype; P: parity; LS: lamb sex; BT: birth type; BW: birth weight; TCAs: total cotyledon area; CN: number of cotyledons; PWs: placenta weight.

### 3.4 General cotyledon characteristics in Morkaraman sheep

General characteristics of the cotyledons in Morkaraman sheep are presented in Table 11. The values obtained in our study found that the GH genotype had a significant ( p<0.01 ) effect on the total cotyledon area, cotyledon weight, and cotyledon density. Furthermore, it was determined that parity had a significant ( p<0.01 ) effect on cotyledon weight. The findings of our study demonstrate that the values obtained in our examination of parity exert a significant effect on the number of cotyledons and the activity of cotyledons ( p<0.05 ). The findings of the study demonstrated that the total cotyledon area and cotyledon density had a significant effect ( p<0.01 ) on the sex of lambs born from Morkaraman sheep. In addition, cotyledon length, cotyledon width, number of cotyledons, cotyledon weight, and cotyledon density were found to have a significant ( p<0.05 ) effect. In the study where the birth weight of male lambs was determined to be higher than the birth weight of female lambs, it was determined that total cotyledon area, cotyledon length, cotyledon width, and cotyledon weight were higher in male lambs. It was determined that the number and density of cotyledons had a significant ( p<0.01 ) effect on the type of birth, and the weight of the cotyledons was also found to have a significant ( p<0.05 ) effect.

### 3.5 Values of cotyledon characteristics according to size in Morkaraman sheep

Values obtained in our studied GH genotype had a significant ( p<0.01 ) effect on medium cotyledon area and medium cotyledon length and had a significant ( p<0.05 ) effect on medium cotyledon width (Table 12). For these parameters, higher figures were reached in the AA genotype than in the AB genotype. It was determined that parity had a significant ( p<0.05 ) effect on medium cotyledon area. It was determined that the values found in terms of midcotyledon area were similar in the third and fourth parity of ewes and lower in those in the second parity. While the effect of lamb sex on midcotyledon area and midcotyledon length was significant ( p<0.05 ), it was determined that lamb sex had a highly significant effect on midcotyledon width ( p<0.01 ). It was determined that male lambs had relatively higher values than female lambs in terms of small cotyledon area, small cotyledon width, and large cotyledon width. It was determined that birth type had a statistically significant ( p<0.01 ) effect on large cotyledon area, large cotyledon width, and large cotyledon length. When these parameters were examined, it was determined that twin lambs had higher values than single lambs.

**Table 12:** Values of cotyledon characteristics according to size in Morkaraman sheep.

	Small	Medium	Large	Small	Medium	Large	Small	Medium	Large
	cotyledon	cotyledon	cotyledon	cotyledon	cotyledon	cotyledon	cotyledon	cotyledon	cotyledon
	area	area	area	length	length	length	width	width	width
Genotype	ns	**	ns	ns	**	ns	ns	*	ns
AA	1.76±0.16	4.42±0.1	7.69±0.16	1.70±0.07	2.61±0.03	7.69±0.16	1.38±0.08	2.21±0.03	2.85±0.04
AB	2.02±0.13	4.16±0.1	7.41±0.21	1.80±0.06	2.52±0.03	7.41±0.21	1.50±0.06	2.16±0.03	2.78±0.05
Parity	ns	*	ns	ns	ns	ns	ns	ns	ns
2	1.87±0.13	4.15 ${}^{B}\pm 0.10$	7.50±0.21	1.75±0.06	2.51±0.03	3.46±0.05	1.41±0.06	2.15±0.03	2.83±0.05
3	1.96±0.15	4.35 ${}^{A}\pm 0.10$	7.59±0.17	1.77±0.06	2.58±0.03	3.53±0.04	1.46±0.07	2.20±0.03	2.78±0.04
4≥	1.85±0.24	4.38 ${}^{A}\pm 0.13$	7.57±0.29	1.72±0.10	2.61±0.04	3.48±0.07	1.44±0.11	2.21±0.04	2.83±0.06
Lamb sex	ns	*	ns	ns	*	ns	ns	**	ns
Female	1.84±0.16	4.19±0.10	7.74±0.18	1.76±0.07	2.54±0.03	3.52±0.04	1.40±0.07	2.15±0.03	2.85±0.04
Male	1.95±0.14	4.40±0.10	7.37±0.21	1.74±0.06	2.59±0.03	3.45±0.05	1.48±0.07	2.22±0.03	2.78±0.05
Birth type	ns	ns	**	ns	ns	**	ns	ns	**
Twin	1.88±0.24	4.41±0.18	8.27±0.25	1.76±0.11	2.60±0.05	3.65±0.06	1.43±0.11	2.21±0.05	2.93±0.05
Single	1.91±0.08	4.18±0.04	6.83±0.14	1.73±0.03	2.54±0.01	3.33±0.03	1.45±0.04	2.16±0.01	2.70±0.03

* Significant ( p<0.05 ). ** Very significant ( p<0.01 ); ns: insignificant. ^A,B^ The difference between means indicated by the same letter between columns is insignificant. Small cotyledon: <20 mm; medium cotyledon: 20–30 mm; large cotyledon: >30 mm.

## 4 Discussion

**Figure 1:**
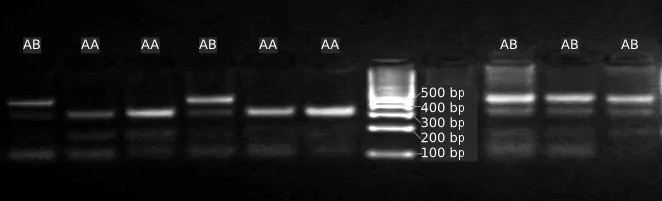
Electrophoretic profile for the GH fragments amplified by PCR in a 2 % ( w/v ) (5 V cm^−1^) agarose gel. A strand with 422 bp (Lanes 1 to 4) or 116 bp (Lanes 5 to 8) appeared to represent amplicons using primer GHF1/GHR1 respectively. AA represented a marker with 100 bp DNA ladder (1500, 1000, 900, 800, 700, 600, 500, 400, 300, 200, 100 bp).

As a result of the study, AA and AB genotypes were identified. BB genotype was not found in the study. In terms of allele frequencies, the frequency of allele A was higher than the frequency of allele B. The GH genotype and allele frequencies obtained in the study are presented in Table 4 in order of lactation. The GH genotype frequency values obtained from the studies were similar to those reported in various breeds: Tibetan (Jia et al., 2014); Matou (Zhang et al., 2011); Salsk (Gorlov et al., 2017); Donggala (Malewa et al., 2014); Kilakarsal and Vembur (Seevagan et al. (2015); Chinese, Black Tibetan, and Gaoyuan (Han et al., 2016); Osmanabadi and Sangamneri (Wickramaratne et al., 2010); Barki, Damascus, and Zaraibi (Mahrous et al., 2018); Harnali (Kumar et al., 2024); and Dorper (Madikadike et al., 2024). In terms of genotype frequency values, the results found in the study by Othman et al. (2015) were found to be lower in terms of AA genotype and higher in terms of AB genotype in Barki and Rahmani breeds (Fig. 1).

Similar to the presence of two genotypes results obtained in our study, Kumar et al. (2024) in Harnali breed and Özay (2019) in Kıvırcık breed found only AA and AB genotypes. They reported that the BB genotype was not found, indicating that the population is not in equilibrium. Madikadike et al. (2024) in their study on Dorper sheep found two genotypes similar to our study, but according to their Hardy–Weinberg test results, they found that their population is in equilibrium. In a study on local Hair goats, Mete (2016) stated the reason why the population was not in equilibrium is due to the selection applied in the herd. In a study conducted by Ouchar (2019), the populations of Ankara, Kıl, and Aleppo goats were found to be in Hardy–Weinberg equilibrium, while the populations of Honamlı, Kilis, and Saanen goats were not in equilibrium. As a result of our study, the results were mostly similar to the literature in terms of population in Hardy–Weinberg equilibrium. This is thought to be due to the fact that the matings in the animals used in the study were not dependent on chance and that the maternal and paternal lines in the herd were mated by selection.

The results revealed that GH genotype had no statistical effect on birth weight, though it was observed that the birth weight of lambs born from ewes with AB genotype was relatively higher than lambs born from ewes with AA genotype. Similar to the results obtained in the study, Abdelmoneim et al. (2017) reported that the birth weights of ewes with AA and AB genotypes were similar. Unlike the results obtained, Hua et al. (2009), Gorlov et al. (2017), Özay (2019), Malewa et al. (2014), and Mete (2016) stated that the birth weight of dam individuals with AB genotype was higher than AA genotype individuals.

The findings obtained in the study examining the effect of genotype on weaning weight are similar to those reported by Gorlov et al. (2017) in Edilbay, Klamyk, and Salsk sheep; Malewa et al. (2014) in Donggala sheep; Hua et al. (2009) in Boer goats; and Mete (2016) in domestic Hair goats. In these studies, weaning weights of lambs with AB genotype were reported to be superior to AA genotype individuals. Unlike the results obtained in this study, Özay (2019) reported that there was no effect of genotype in Kıvırcık sheep. The daily weight gain obtained in our study was higher than that reported by Hua et al. (2009), Udum (2009), Gorlov et al. (2017), and Malewa et al. (2014) and lower than that reported by Özay (2019) and El-Mansy et al. (2023).

In many studies reported in the literature on birth weight, daily live weight gain, and weaning weight, it has been observed that the breeds used in the studies have similar results despite the differences in care feeding and climatic conditions, and AB-genotyped individuals have superior growth-development characteristics compared to AA-genotyped individuals.

Similar to the results obtained in this study in terms of birth weight averages of lambs born in different years from Morkaraman ewes, Kandemir et al. (2021) reported that the highest birth weight was reached in the fourth lactation and the lowest in the second lactation. Bağcı (2021) reported the highest in the fourth lactation and the lowest in the first year of life. Özyürek and Türkyılmaz (2020) reported the highest in the fourth lactation and the lowest in the sixth lactation. On the other hand, the values reported for sex and type of birth were similar to Mete (2016) and Özyürek (2019), and Ocak et al. (2014) reported that both parameters had no effect on birth weight, and Özyürek and Türkyılmaz (2020) stated that sex had a significant effect, while type of birth had no effect.

The higher birth weights observed in lambs born during the second year may be attributed not only to annual variations in climatic conditions, pasture quality, and feed composition, but also to physiological factors such as improved uterine capacity, better placental efficiency, and enhanced maternal body condition in ewes of higher parity. It is well established that a higher number of parities in ewes, particularly those in their second or subsequent parities, often result in greater reproductive tract maturity and improved nutrient partitioning capacity. This, in turn, can positively influence fetal development and birth weight. Consequently, the elevated weaning weights recorded in the second year are likely a combined result of these increased birth weights and the improved maternal support for postnatal growth. It is a well-documented fact that multiparous ewes, particularly those in their second or subsequent parities, often exhibit greater reproductive tract maturity and improved nutrient partitioning capacity. These factors can positively influence fetal development and birth weight. Consequently, the elevated weaning weights recorded in the second year are likely a combined result of these increased birth weights and the improved maternal support for postnatal growth.

Similar to the placenta weights obtained in terms of parity in the study, Ocak et al. (2013), Kandemir et al. (2021), and Bağcı (2021) did not report a significant difference. Contrary to our results, Özyürek and Türkyılmaz (2020) reported that ewes in the second lactation had lower placenta weights, while Konyalı et al. (2007) reported that ewes in the third lactation had higher placenta weights.

Similar to the results obtained in terms of placental efficiency, Konyalı et al. (2007), Ocak et al. (2013), Özyürek (2019), and Özyürek and Türkyılmaz (2020) reported that the effect of parity was insignificant. However, Wallace et al. (2002), Echternkamp (1993), and Madıbela (2004) stated that placental efficiency increased with parity.

Similar to the birth weight findings, the effect of AB genotype in terms of placenta characteristics was found to be statistically insignificant; however, it was observed to have relatively higher values than the AA genotype. It is thought that the higher birth weights of the ewes with AB genotype also affect the calculated placenta characteristics. The placenta area obtained in our study for male and female lambs was higher than that reported by Brzozowska et al. (2020), Özyürek and Türkyılmaz (2020), and Şen et al. (2021) and lower than that reported by Olfati et al. (2016), Yan et al. (2018), Kandemir et al. (2021), Bağcı (2021), Şen and Kaya (2022), and Cantou et al. (2024).

Similar to birth weights, it was determined that male lambs had higher values than female lambs in terms of placental efficiency, but this difference was not statistically significant. Kaya (2020) reported that the gene expression level of IGF-I and IGF-II hormone, which is another hormone that affects growth and development characteristics in addition to GH hormone, has an effect on growth and development in ewes. Authors observed that the amount of IGF hormone release was higher in ewes with twin pregnancies. The results found in the study are similar to the values reported by Özyürek (2019), Özyürek and Türkyılmaz (2020), and Konyalı et al. (2006) but differ from the values reported by Bağcı (2021).

The values obtained as a result of the study in terms of the values determined on the placenta weight of birth type are similar to the values reported by Vonnahme et al. (2008), Özyürek (2019), Özyürek and Türkyılmaz (2020), Kandemir et al. (2021), Bağcı (2021), Şen and Kaya (2022), Kaya (2020), Ocak et al. (2013), and Ocak et al. (2009). The values obtained in the study, in which it was determined that the placenta birth type of single lambs had no effect, are similar to the values reported by Özyürek (2019), Özyürek and Türkyılmaz (2020), and Kandemir et al. (2021). According to the results obtained in the study, the total cotyledon area was found to be lower than the values reported by Kandemir et al. (2021), Bağcı (2021), and Şen et al. (2021) and higher than the values reported by Ocak et al. (2013) and Kaya (2020). The total cotyledon area obtained in our study is lower than the values reported by Özyürek and Türkyılmaz (2020). The reason for the difference is thought to be due to the differences in factors such as age, parity, breeding system, and genotype of the ewes used in the studies. In studies reporting that higher values were reached in terms of cotyledon number and cotyledon weight in the third parity of ewes compared to other parities, the results were similar to the values reported by Ocak et al. (2013) and Konyalı et al. (2007), while they differed from the values reported by Kandemir et al. (2021) and Bağcı (2021). The results obtained in terms of parity were found to be similar to the values reported by Özyürek and Türkyılmaz (2020) in terms of cotyledon density and cotyledon number in the Morkaraman breed but lower than the values reported in terms of cotyledon efficiency. It is thought that the differences that emerged in the study conducted in the same breed and investigating similar parameters may have arisen from the differences in climate, feed material used in the study, and maintenance and feeding conditions.

The cotyledon area of twin lambs obtained in the study was lower than that reported by Kandemir et al. (2021), Bağcı (2021), and Şen et al. (2021) and higher than the values reported by Ocak et al. (2013) and Kaya (2020). The total cotyledon area obtained in our study was lower than the values reported by Özyürek (2019) and Özyürek and Türkyılmaz (2020). The reason for the difference is thought to be the effects of factors such as age, parity, and breeding systems of the ewes used in the study. The cotyledon length and width values found in our study were higher than those reported by Ocak et al. (2014), Özyürek (2019), Özyürek and Türkyılmaz (2020), Kaya (2020), Şen and Kaya (2022), Urun and Şirin (2023), and Cantou et al. (2024) and lower than those reported by Yan et al. (2018) and Şen et al. (2021).

There is a highly significant interaction between genotype and parity in terms of cotyledon length and cotyledon efficiency and a significant interaction in terms of cotyledon width and cotyledon weight. There was a significant interaction between genotype and birth type in terms of cotyledon weight and a highly significant interaction in terms of cotyledon number, cotyledon efficiency, and cotyledon density. There was an interaction between parity and sex for all cotyledon characteristics. There was a highly significant interaction between parity and birth type in terms of total cotyledon area and a significant interaction in terms of cotyledon weight and cotyledon efficiency. There was a significant interaction between birth type and sex in terms of cotyledon number and cotyledon efficiency. There was a significant interaction between genotype and sex in terms of general cotyledon characteristics.

## 5 Conclusion

The growth characteristics of the GH gene polymorphisms identified in Morkaraman ewes were analyzed, and it was determined that the ewes with the AB genotype exhibited superior growth characteristics and placental characteristics. Similarly, an analysis of lambs born in different years from ewes with the AB genotype revealed that they exhibited enhanced growth characteristics. In light of these findings, it may be feasible to identify individuals with specific genotypes that exhibit enhanced growth characteristics associated with GH gene polymorphisms, which can be discerned from birth. It is thought that the population is not in equilibrium since the matings in the animals used in the study, in which the population Hardy–Weinberg equilibrium was found to be similar to the literature reports, are not dependent on chance and there is selection in the herd. In light of the growing body of genetic research on ovine animals in recent years, it is believed that the existing gaps in the literature can be addressed by examining different gene regions, different breeds, and larger populations. Consequently, in addition to the conventional breeding techniques, the latest breeding techniques can be employed more extensively by utilizing genetic parameters.

## Data Availability

The original data used in this study are available from the corresponding author upon request.
